# Excluding Echo Shift Noise in Real-Time Pulse-Echo Speed-of-Sound Imaging

**DOI:** 10.3390/s23125598

**Published:** 2023-06-15

**Authors:** Parisa Salemi Yolgunlu, Naiara Korta Martiartu, Urs Richard Gerber, Martin Frenz, Michael Jaeger

**Affiliations:** Institute of Applied Physics, University of Bern, Sidlerstrasse 5, 3012 Bern, Switzerlandmartin.frenz@unibe.ch (M.F.)

**Keywords:** pulse-echo ultrasound, speed of sound, phase noise, echo shift, inverse problem

## Abstract

Computed ultrasound tomography in echo mode (CUTE) allows real-time imaging of the tissue speed of sound (SoS) using handheld ultrasound. The SoS is retrieved by inverting a forward model that relates the spatial distribution of the tissue SoS to echo shift maps detected between varying transmit and receive angles. Despite promising results, in vivo SoS maps often show artifacts due to elevated noise in echo shift maps. To minimize artifacts, we propose a technique where an individual SoS map is reconstructed for each echo shift map separately, as opposed to a single SoS map from all echo shift maps simultaneously. The final SoS map is then obtained as a weighted average over all SoS maps. Due to the partial redundancy between different angle combinations, artifacts that appear only in a subset of the individual maps can be excluded via the averaging weights. We investigate this real-time capable technique in simulations using two numerical phantoms, one with a circular inclusion and one with two layers. Our results demonstrate that the SoS maps reconstructed using the proposed technique are equivalent to the ones using simultaneous reconstruction when considering uncorrupted data but show significantly reduced artifact level for data that are corrupted by noise.

## 1. Introduction

Quantitative ultrasound (US) imaging of tissue speed of sound (SoS) has attracted considerable attention for two main reasons. First, the SoS depends on the density and compressibility of the medium in which the US waves propagate, meaning that SoS varies in tissues with different compositions and microarchitectures. Thus, we can use this property for identifying disease-related changes in these tissue properties. For instance, SoS has proven clinically relevant in diagnosing and grading hepatic steatosis [1,2], differentiating malignant and benign solid breast lesions [3,4,5], and quantifying muscle loss [6,7,8]. Second, the B-mode image quality suffers from aberration artifacts due to the disagreement between the true SoS and the (typically constant) SoS assumed for beamforming. Knowledge of the tissue’s SoS distribution can be used to correct aberration artifacts [9,10,11].

Several methods have been proposed to reconstruct the SoS in pulse-echo mode [12,13,14,15,16,17]. In this study, we use computed ultrasound tomography in echo mode (CUTE) [18]. CUTE allows for the real-time quantification of the spatial distribution of the tissue’s SoS with promising spatial and contrast resolution [19,20]. In CUTE, we insonify the tissue under various transmit (Tx) angles and record the corresponding radio frequency (RF) signals for varying receive (Rx) angles. The recorded US echoes are beamformed using the delay-and-sum method, assuming a uniform SoS. Since tissue is inherently heterogeneous, echoes contain aberration delays, i.e., a mismatch between the anticipated and actual round-trip time of US propagation, resulting in a bias of the reconstructed position. This bias varies with changing Tx and Rx angles, according to the spatial distribution of the SoS, leading to a spatial shift that can be quantified as an echo phase shift. By assuming that US waves propagate as straight rays, we can linearly relate these echo shifts to the spatial distribution of slowness deviation, namely the difference between the inverse of the true SoS and the inverse of the assumed SoS, and solve the corresponding inverse problem to retrieve SoS maps.

**Figure 1:**
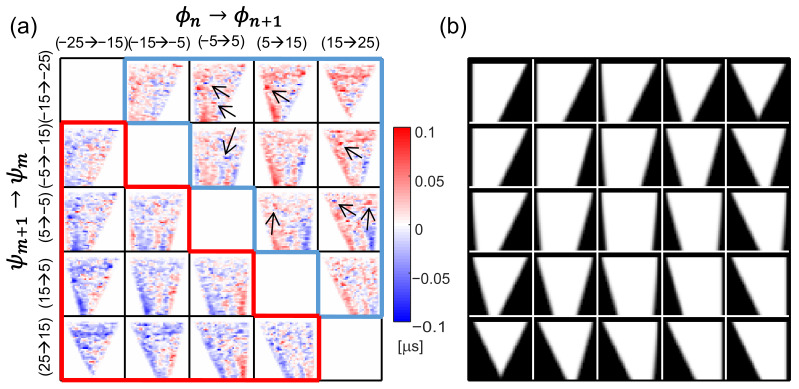
(**a**) In vivo example of echo shift maps for different combinations of the transmit angle $\phi_{n}$ and receive angle $\psi_{m}$. (**b**) Weight masks are used to exclude pixels where echo shift information is missing in (**a**) due to the limited probe aperture for the different transmit/receive angles. Values range from one (full information) to zero (no information).

CUTE has shown promising results in tissue-mimicking phantoms and in vivo [19,21]. In vivo echo shift data are, however, often marked by systematic elevated noise that can arise due to attenuation, aberration, and reverberations, among others, introducing artifacts in the SoS map. This elevated noise is typically marked by a relatively small number of sparse outliers from an otherwise rather smooth echo shift distribution. An example of in vivo echo shift data is shown in Figure 1a, where a few outliers are marked by arrows. Very importantly, the elevated noise has a different spatial distribution for different angle combinations.

Since the data in the different echo shift maps are partially redundant, there is potential in reducing artifacts by defining areas with sparse elevated noise as missing data areas and complementing missing data in one map with information obtained in another map. One approach to do this is to exclude missing data areas from the forward model, similar to how it is already conducted for excluding areas of missing echoes due to the limited probe aperture size [16,22]. In a real-world scenario where the echo shift noise changes while scanning the tissue with the US probe, this approach requires recomputing the forward and inverse operators for every new probe location. This recomputation is time-consuming and limits the achievable SoS image frame rate.

In this study, in order to overcome this problem, we propose an alternative approach where we reconstruct an individual SoS map for each echo shift map separately and then perform a weighted average over all SoS maps to obtain the final SoS map, as opposed to combining all echo shift data in the forward/inverse problem simultaneously. A weighted average allows us to exclude areas that are corrupted in one map but not in others. This approach is computationally efficient as the inverse operators for reconstructing the individual SoS maps from the individual echo shift maps can be computed once, and only the averaging weights need to be recomputed for each probe position, thus substantially reducing the computation time. The paper is organized as follows. In Section 2, we revise the established implementation of CUTE and present the proposed technique for SoS reconstruction. In Section 3, we describe the simulation framework that we used for this study, and we present the results for two exemplary digital phantoms mimicking a circular inclusion and layered tissues in Section 4. For both digital phantoms, we prove that the reconstructed SoS map—by using the proposed SoS reconstruction technique—is equivalent in terms of contrast and noise level to the reconstructed SoS map in the ideal scenario with minimum echo shift noise. After that, we add synthetic noise to the echo shift maps and illustrate how the proposed technique can provide results with reduced artifacts compared to the previous technique.

## 2. Theory

In this section, we first summarize the echo shift tracking method and the forward model that CUTE relies on (Section 2.1). The reader is referred to [19] for a more detailed explanation of the technical aspects of CUTE. Then, in Section 2.2, we formulate the SoS reconstruction method that CUTE has used so far (reference method). Finally, in Section 2.3, we present our new reconstruction approach.

### 2.1. Echo Shift Tracking

In CUTE, we acquire pulse-echo data to generate complex radio frequency (CRF)-mode maps for different combinations of Tx steering angles $\phi_{n}$ and Rx steering angles $\psi_{m}$. Lateral resolution is provided by weak focusing along these directions. The Tx steering can be achieved by line-by-line scanning, by synthetic aperture imaging, or—as in previous studies—by coherent compounding of plane wave acquisitions. The Rx steering can either be achieved explicitly via angle apodization in the delay-and-sum (DAS) beamforming [23], or, as in previous studies [19,20,22], via spatial frequency domain decomposition after DAS beamforming using the full receive aperture. Finally, we use zero-lag complex cross-correlation to track the spatially resolved echo phase change between CRF-mode maps that have been obtained with different angle combinations. CUTE uses the common-mid-angle [19] principle to make sure that the echoes detected with the different combinations are well correlated, and to avoid echo shift biases that occur due to an implicit Rx angle influence depending on the echo distribution. This is conveniently achieved by choosing ϕ and ψ from the same discrete set of equidistant angles, $\phi_{n},\psi_{m}\in \left\{\phi_{k},k=1\ldots N\right\}$, and by tracking between angle pairs $\left(\phi_{n},\psi_{m+1}\right)$ and $\left(\phi_{n+1},\psi_{m}\right)$, with common mid angles $\gamma =\left(\phi_{n}+\psi_{m+1}\right)/2=\left(\phi_{n+1}+\psi_{m}\right)/2$, resulting in echo echo shifts $\Delta \Theta_{n,m}\left(x,z\right)$. The diagram in Figure 1a shows an example of echo shift maps obtained in vivo. Note that maps that are located symmetrically about the diagonal of this diagram are theoretically identical due to transmitter/receiver reciprocity, apart from a sign resulting from the change in the tracking direction (and, for the same reason, maps along the diagonal of the diagram are zero). Slight differences that exist in practice between these maps are eliminated by taking the average after sign inversion. Finally, only the maps located inside either the blue or the red frames in Figure 1a are used for SoS reconstruction.

The relationship between detected echo phase shifts and aberration delays can be expressed as [19]

(1) $$\Delta \Theta_{n,m}=2\pi f_{0}\left\{\frac{\tau (\phi_{n},\psi_{m+1})}{\cos\left(\frac{1}{2}\left(\phi_{n}-\psi_{m+1}\right)\right)}-\frac{\tau (\phi_{n+1},\psi_{m})}{\cos\left(\frac{1}{2}\left(\phi_{n+1}-\psi_{m}\right)\right)}\right\},$$

where $f_{0}$ is the center frequency and τ(ϕ,ψ) refers to the sum of the aberration delay in transmission and receive. For simplicity, we omitted the coordinate (x,z) from the equation. By assuming straight-ray propagation, the aberration delay is given by the sum of line integrals of slowness deviation Δσ (the difference between the actual slowness and the slowness assumed for DAS) along the Tx and Rx paths determined by the transmit and receive setting (n,m), i.e.,

(2) $$\tau \left(\phi_{n},\psi_{m},x,z\right)=\int_{\phi_{n}}^{\left(x,z\right)}\;\Delta \sigma \left(x^{\prime },z^{\prime }\right)dl+\int_{\psi_{m}}^{\left(x,z\right)}\;\Delta \sigma \left(x^{\prime },z^{\prime }\right)dl.$$

Integral limits indicate that line integrals are performed along the indicated angle, starting at the aperture surface and ending at the point (x,z). By defining Equations (1) and (2) on discrete coordinate grids, we can express the combined forward model using matrix notation as

(3) $$\Delta \Theta_{n,m}=L_{n,m}\Delta \sigma ,$$

where $L_{n,m}$ denotes the forward operator, $\Delta \Theta_{n,m}$ is a vector containing the phase shift values, and Δσ represents the vectorized slowness deviation values. Each row in the forward operator $L_{n,m}$, when multiplied with the vector Δσ, generates the echo shift value corresponding to one pixel in $\Delta \Theta_{n,m}$. To account for pixels where echo shift data are missing, the corresponding row in $L_{n,m}$ is set to zero. This approach is used to exclude pixels inside the areas where echoes cannot be detected for the given Tx/Rx angles due to the limited aperture sizes (the Tx/Rx shadows). Figure 1b illustrates the masks that are used to define these areas for the same angle combinations used in Figure 1a.

### 2.2. Reference Method

The final step of CUTE is to reconstruct Δσ from the $\Delta \Theta_{n,m}$. This is typically done by formulating a least-squares inverse problem based on Equation (3). Since the angle range is limited in pulse-echo imaging, this inverse problem is ill-posed and requires regularization to obtain meaningful solutions. Following [19], we consider the first-order Tikhonov regularization. The solution to this regularized inverse problem can then be expressed as

(4) $$\Delta \sigma =\Pi^{-1}\sum_{n=1}^{N-1}\sum_{m<n}^{M-1}L_{n,m}^{T}\Delta \Theta_{n,m},$$

where the posterior covariance matrix $\Pi^{-1}$ is given by

(5) $$\Pi =\sum_{n=1}^{N-1}\sum_{m<n}^{M-1}L_{n,m}^{T}L_{n,m}+\lambda_{1}D_{x}^{T}D_{x}+\lambda_{2}D_{z}^{T}D_{z}.$$

The superscript *T* denotes the matrix transpose operation, *N* is the total number of Tx angles, and *M* is the total number of Rx angles. $D_{x}$ and $D_{z}$ are first-order finite-difference operators in *x* and *z* directions, respectively, that enforce smooth solutions, and $\lambda_{1}$ and $\lambda_{2}$ are the corresponding regularization parameters. Finally, the reconstructed SoS is recovered from the reconstructed slowness deviation Δσ according to

(6) $$c={\left(\Delta \sigma +\frac{1}{c_{0}}\right)}^{-1},$$

where $c_{0}$ is the assumed SoS for beamforming.

In the method presented in this section (henceforth called the reference method), the inversion considers all echo shift maps simultaneously to reconstruct the SoS map. This allows us to make use of data redundancies that exist between the different echo shift maps because they are all generated by the same SoS distribution: by combining echo shift data that were obtained with different angle combinations, the impact of echo shift noise is reduced.

The reference method implicitly assumes independently and identically normal distributed noise. It is, thus, sub-optimal in a situation where echo shift data are noisier inside discrete areas. In this case, one solution for reducing artifacts is to exclude such noisy areas explicitly in the forward operator, by setting rows of the $L_{n,m}$ to zero that correspond to pixels within the noisy data areas in the echo shift maps. The disadvantage of this approach is that it encodes geometric information of tissues into the forward operator. It, thus, becomes dependent on the probe location, and the posterior covariance matrix $\Pi^{-1}$ needs to be newly calculated for each location while scanning the tissue. This greatly limits either the achievable frame rate or the pixel resolution of the SoS map.

### 2.3. Proposed Method

To overcome this problem, we suggest reconstructing an individual SoS map per echo shift map and then perform a weighted average to generate the final result. This approach allows us to discard areas in SoS maps corresponding to noisy echo shift data while providing real-time solutions. The model inversion per echo shift map is formulated as

(7) $$\Delta \sigma_{n,m}=\Gamma_{n,m}^{-1}L_{n,m}^{T}\Delta \Theta_{n,m},$$

where

(8) $$\Gamma_{n,m}=L_{n,m}^{T}L_{n,m}+\eta_{1}D_{x}^{T}D_{x}+\eta_{2}D_{z}^{T}D_{z}.$$

The individual SoS maps are obtained as

(9) $$c_{n,m}={\left(\Delta \sigma_{n,m}+\frac{1}{c_{0}}\right)}^{-1}.$$

As before, $\eta_{1}$ and $\eta_{2}$ denote regularization parameters, which can differ from the ones in Equation (5) due to the different nature of the inverse problem. In this work, the regularization parameter values are chosen to ensure that the two techniques provide the same spatial resolution, enabling a fair comparison of the artifact level. In order to calculate an SoS map *c* that combines the information from all Tx/Rx angles, we perform a weighted average over all $c_{n,m}$, as

(10) $$c=\frac{\sum_{n=1}^{N-1}\sum_{m<n}^{M-1}W_{n,m}c_{n,m}}{\sum_{n=1}^{N-1}\sum_{m<n}^{M-1}W_{n,m}},$$

where $W_{n,m}$ are masks that are used to exclude the map areas that do not contain meaningful information due to the noise in the echo shift maps.

## 3. Materials and Methods

### 3.1. Echo Shift Simulation

We used a home-built framework based on Matlab^®^ (MathWorks, Inc., MA, USA) to simulate echo US signals acquired with a linear array probe from digital phantoms. A digital phantom is characterized by a real-valued 2D distribution of ground-truth SoS together with a 2D complex-valued distribution of “echogenicity”. The hybrid angular spectrum (or Fourier split-step) technique [24,25] is used to simulate the complex-valued Green’s function for each transducer element, accounting for the ground-truth SoS distribution of the digital phantom. Based on these Green’s functions, a full-matrix capture is simulated by assuming the first-order Born approximation: for each pair of transmitting and receiving elements, the product of the Green’s functions of the respective elements with the echogenicity is integrated over the 2D plane. This is repeated for a variety of frequencies covering the probe bandwidth, and the results are combined in a Fourier sum to form the time-domain signal. From the full-matrix capture, we generate signals for plane-wave transmissions at specific angles.

Using this framework, we simulated plane wave acquisitions using a 5 MHz center frequency, 2 MHz bandwidth linear array probe containing 128 elements with a pitch size of 0.3 mm. In agreement with earlier in vitro and in vivo studies (the reader is referred to Stähli et al. [19,20] for a detailed description), we chose Tx angles ranging from $-27.5^{\circ }$ to $27.5^{\circ }$ in $0.5^{\circ }$ steps. For each angle, we generated a CRF-mode map using DAS over the full receive aperture. From these maps, we generated coherently compounded CRF-mode maps for Tx angles ranging from $-25^{\circ }$ to $25^{\circ }$ in $5^{\circ }$ steps, with an angular aperture per Tx angle of $\pm 2.5^{\circ }$. To implement the common mid-angle principle, the Tx-only steered CRF-mode maps were decomposed into Tx-Rx steered maps using k-space filtering with a filtering angular aperture of $2.5^{\circ }$. The echo shift was quantified via the phase angle of the complex correlation using an axial correlation window length of 2 mm. The echo shifts obtained with $5^{\circ }$ steps were summed over successive steps to generate echo shift maps for Tx-Rx angles ranging from $-25^{\circ }$ to $25^{\circ }$ in $10^{\circ }$ steps. For this study, we exemplarily simulated echo shift data for two digital phantoms, one with a circular compartment with 20 m/s SoS contrast inside a uniform background, mimicking tumor imaging, and one built from two compartments with 65 m/s contrast across a horizontal interface, mimicking liver imaging. The SoS inside the compartments was chosen to be uniform to minimize aberrations and, thus, keep aberration-related echo shift noise to a minimum. The echogenicity was chosen to be independently and identically normally distributed to minimize noise related to echogenicity. Because only first-order echoes are simulated (the first-order Born approximation), artifacts caused by higher-order (multipath) echoes are avoided by design. Altogether, these simulations allow studying the performance of CUTE in an idealized scenario with a minimum amount of noise.

### 3.2. Synthetic Noise

The simulations were used to provide the ground truth of SoS image quality that can be achieved in an ideal scenario with a minimum amount of noise. In reality, additional echo shift noise results from multipath echo clutter, multiple scattering, highly attenuating tissue, and spatial variations of echogenicity. This noise is marked by a relatively small number of sparse outliers from an otherwise smooth echo shift distribution. To imitate this noise, we added/subtracted values 0.1 μs to randomly selected pixels inside circular areas (radius 10 mm) with a randomly selected center. Compared to generating the noisy echo shift via a more realistic simulation, adding synthetic noise has a big advantage for the statistical analysis of results: it allows us to combine the same echo shift data with a large variety of noise realizations without having to repeat the US simulation. This ensures optimal comparability of the results. For the purpose of illustrating the proposed method, we added noise only to a random selection of five out of the ten echo shift maps. This greatly simplifies the didactic take-home message of our study because it allows us to remove noise by fully removing a small number of selected maps as opposed to removing a large number of limited areas.

## 4. Results

This section is divided into two parts. In the first part (Section 4.1), we employ both methods to obtain the SoS map for both phantoms from echo shift maps without additional noise, including all available echo shift data. Here, we want to determine whether the proposed method has a disadvantage compared to the reference method in terms of the SoS artifact level. Note that there is a trade-off between the spatial resolution and artifact level. For a fair comparison of the SoS artifact level, we adapt the regularization parameters of the two methods so that they show equal spatial resolution. Even in the most ideal scenario, a minimum level of phase noise occurs due to the randomness of the US speckle. For this reason, we investigate the influence of random artifacts due to speckle by calculating the per-pixel mean and standard deviation of the reconstructed SoS over ten simulations of the same SoS distribution but with different realizations of the random echogenicity distribution. The same echogenicity realizations were used with both digital phantoms. In the second part (Section 4.2), we use both methods with the adapted regularization weights to reconstruct SoS maps from echo shift maps where synthetic noise was added. Here, we use the echo shift data obtained with a single speckle realization but generate 1000 synthetic noise realizations in order to analyze the influence of the additional noise on the artifact level. The same noise realizations were used with both digital phantoms to ensure the best comparability of results.

### 4.1. Minimum Noise SoS Reconstructions

**Figure 2:**
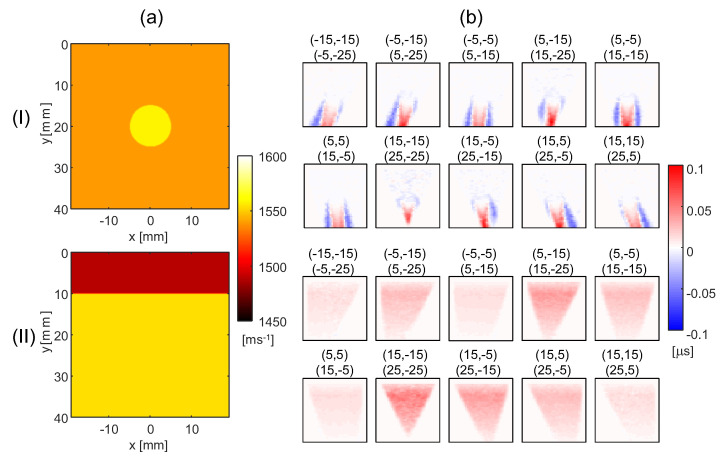
(**a**) Ground-truth speed-of-sound (SoS) maps for the circular inclusion phantom (I) and the layer phantom (II). The SoS value inside the circular area and background is 1560 m/s and 1540 m/s, respectively. The SoS value for the upper and lower layer is 1490 m/s and 1555 m/s, respectively. (**b**) The detected echo shift maps for the circular inclusion phantom (I) and layer phantom (II). The angle pairs $\left(\phi_{n},\psi_{m+1}\right)\to \left(\phi_{n+1},\psi_{m}\right)$ with angles $\phi_{n},\psi_{m}\in -25^{\circ }$:$10^{\circ }$:$25^{\circ }$ are denoted on top of the corresponding individual maps.

The ground-truth SoS distribution of the circular inclusion and two-layer phantom is shown in Figure 2a. The detected echo shift maps with no additional noise are exemplarily shown for one echogenicity realization in Figure 2b. Note that we only show the subset of echo shift maps that is actually used for the SoS reconstruction, corresponding to the upper triangle in Figure 1a.

**Figure 3:**
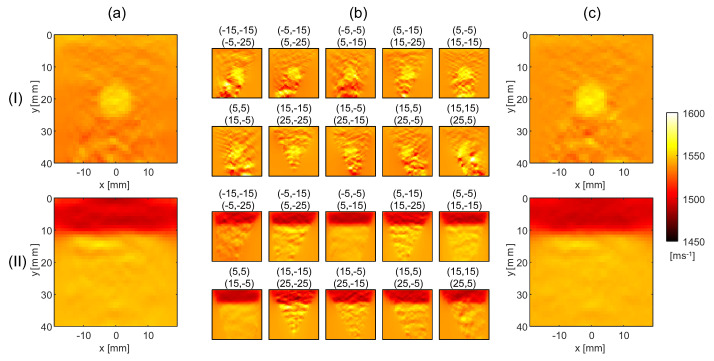
(**a**) Reconstructed SoS maps using the reference method, (**b**) SoS maps reconstructed for the individual echo shift maps shown in Figure 2b, and (**c**) SoS maps when averaging the individual maps in (**b**), for the circular inclusion phantom (I) and the layer phantom (II).

The reconstructed SoS maps are defined on a rectilinear grid with lateral and axial dimensions of 40 mm by 40 mm, respectively, with a grid spacing of 1 by 1 mm. For the reference method, we use regularization parameters $\lambda_{1}=1.25$ and $\lambda_{2}=1.75$, and for the proposed method, $\eta_{1}=0.361$ and $\eta_{2}=0.0361$. Figure 3a exemplarily shows the SoS maps obtained with the reference method using the echo shift data in Figure 2b for the two phantoms.

As explained earlier, the first step in the proposed method is to reconstruct an individual SoS map from each echo shift map. Figure 3b shows these maps exemplarily for the echo shift maps in Figure 2b. Note that the circular inclusion and the layer structure are visible in each SoS map. This highlights the partial redundancy of the different echo shift maps and corresponding SoS maps. Even though the artifact level (observed as variations in SoS inside the uniform phantom compartments) is similar to the contrast of the circular inclusion for certain angle combinations, the artifacts appear different on the different maps. It is for this reason that combining multiple tracking angle pairs is beneficial for reducing artifacts. In the reference method, this is achieved by the simultaneous SoS reconstruction from all maps. In the proposed method, the final SoS map is computed by a weighted average over the individual SoS maps. Figure 3c shows the resulting SoS maps for the two phantoms.

Note that, in the individual maps in Figure 3b, SoS values are artificially enforced by regularization inside the areas where echo shift data are missing due to the Tx/Rx shadows seen in Figure 1b. Thus, the choice of the averaging weights $W_{n,m}$ in these areas influences the spatial distribution of the regularization strength in the average SoS map and, in turn, the artifact level. We empirically determined that the artifact level in the proposed method matches the reference method when the $W_{n,m}$ are set as the average between one and the value of the masks shown in Figure 1b.

**Figure 4:**
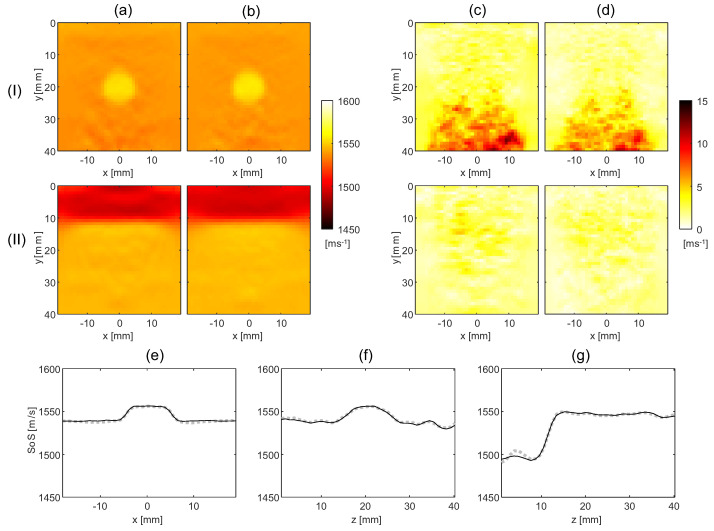
Results of the statistical analysis over 10 speckle realizations; (**a**,**b**) are the average SoS maps for the reference and proposed methods, respectively, and (**c**,**d**) are the standard deviation maps for the two methods, for the circular inclusion phantom (I) and the layer phantom (II). (**e**,**f**) show the lateral and axial profiles through the average SoS maps of the circular inclusion phantom, for reference (dashed gray) and the proposed method (solid black). The profiles intersect at the center of the inclusion; (**g**) shows the axial profiles through the layer phantom located at x=0 for the reference (dashed gray line) and the proposed method (solid black).

Comparing Figure 3c with Figure 3a, both methods reconstruct the SoS in the circular inclusion and layer phantom with a similar artifact level. In this scenario the noise is related to US speckle. To investigate the level of speckle-related artifacts, the average and standard deviation of SoS maps over ten speckle realizations are shown for the two phantoms for both techniques in Figure 4a,b.

The standard deviation maps show that, apart from inside an area below the circular inclusion, the artifact level due to speckle is around 3 m/s. The substantially larger standard deviation below the circular inclusion shows the interplay between aberrations and the reflector distribution in generating speckle-related artifacts. Notably, the artifact level in the SoS maps resulting from both SoS reconstruction techniques is very similar. This is an interesting result because it demonstrates that there is little disadvantage in using the proposed method compared to the reference method in an ideal situation with only speckle noise. To demonstrate that the above comparison is fair, i.e., that the regularization parameters were chosen in a way that matches the spatial resolution of the two methods, we compare the lateral and axial profiles of the average SoS maps, see Figure 4e–g. These profiles are nearly identical for the two methods, confirming that the previous noise level comparison is meaningful. In conclusion, the proposed method can be used as a technique for SoS reconstruction without drawbacks compared to the reference method.

### 4.2. SoS Reconstruction from Noisy Echo Shift Maps

**Figure 5:**
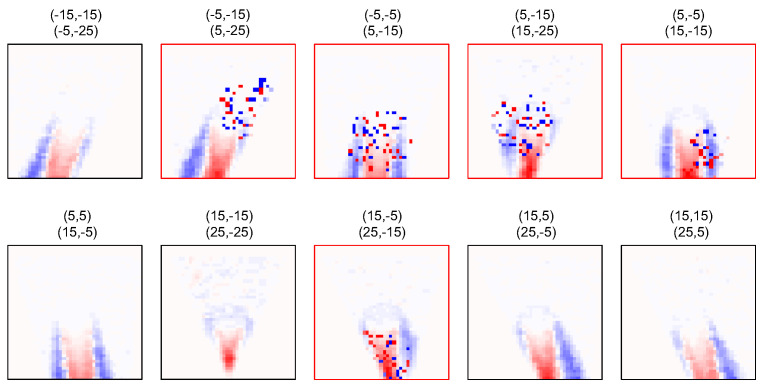
Example echo shift maps with added synthetic noise for the circular inclusion phantom. Five out of ten maps where noise was added are marked by red frames.

**Figure 6:**
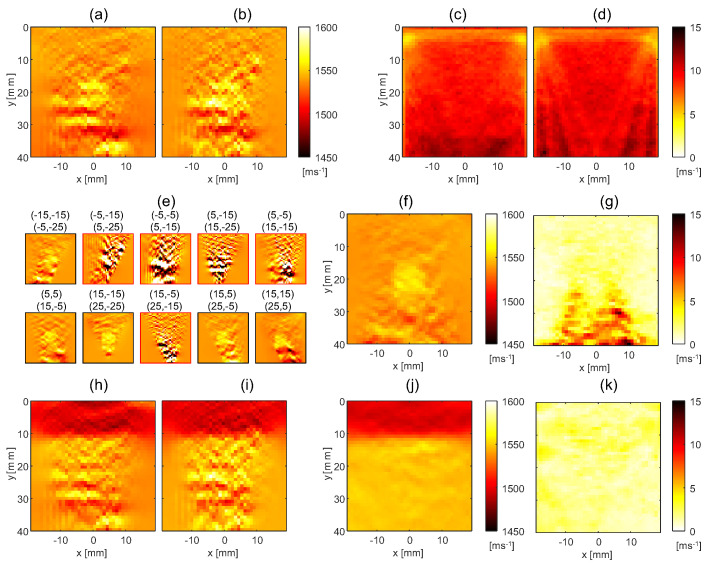
(**a**,**b**) Examples of SoS reconstructions for the circular inclusion phantom obtained with the reference and the proposed method, respectively, using the data shown in Figure 5. (**c**,**d**) Standard deviation maps of reconstructed SoS values over 1000 added noise realizations (but the same speckle realization) using the reference and the proposed method, respectively. (**e**) Individual SoS maps obtained from example echo shift maps shown in Figure 5. Five out of ten individual maps where the noise was added are marked by red frames. (**f**) An example of a reconstructed SoS map obtained with weighted averaging of the individual SoS maps in (**e**). (**g**) The standard deviation map of reconstructed SoS values using the method in (**f**) over 1000 noise realizations. (**h**,**i**) SoS reconstructions for the layer phantom obtained with the reference and the proposed method, respectively, using the same noise realization as in (**a**,**b**,**e**,**f**). (**j**) An example of the reconstructed SoS map obtained with weighted averaging. (**k**) SoS standard deviation map over 1000 noise realizations using the method in (**j**).

In this section, we compare the reconstructed SoS maps for echo shift data with added noise using both methods. This analysis is performed for a single speckle realization in order to highlight the artifacts caused by the added noise alone. Figure 5 shows an example of echo shift maps where noise was randomly added using the method described in Section 3.2 for the circular inclusion phantom. The corresponding SoS reconstructions are shown in Figure 6a,b, using the reference and proposed methods, respectively.

Figure 6a,b show the SoS maps of the circular inclusion phantom reconstructed from the echo shift data shown in Figure 5, when using the reference and the proposed method, respectively, without excluding data. As a result of the added noise, both maps show a high level of artifacts, which makes it difficult to discern the circular inclusion. Figure 6c,d show the standard deviation over 1000 random realizations of added synthetic noise, of the reconstructed SoS value in each pixel for the reference and proposed methods, respectively. Similar to the standard deviation due to speckle (Figure 4c,d), the standard deviation due to randomly added noise is similar for both methods. Figure 6e shows the individual SoS maps reconstructed from the different echo shift maps that were shown in Figure 5. Comparing this figure to the corresponding panel in Figure 3b, strong artifacts are visible in the individual maps where noise was added to the echo shift data. Even though the relation is not one-to-one, these artifacts are roughly localized at the areas where noise was added. This opens up future prospects for identifying noisy areas in the echo shift maps and discarding data corresponding to these areas in individual SoS maps when performing weighted averaging (see the Discussion section). Here, we illustrate the proposed method in a simplified scenario where only part of the echo shift maps are corrupted, and the averaging weights can be chosen so as to fully exclude these SoS maps and use the remaining maps to calculate the final SoS map. The result is shown in Figure 6f. In comparison to Figure 6b, where all maps were used, this SoS map shows a substantially lower artifact level, and the circular inclusion becomes visible. Note that the artifact level is higher than in Figure 3c, where only speckle-related artifacts were present. The reason is that the averaging was performed over a reduced number of individual SoS maps. Figure 6g shows the standard deviation of the SoS values over the 1000 noise realizations. This result demonstrates the strongly reduced artifact level compared to Figure 6d. Interestingly, this map shows a pattern similar to the standard deviation over the different echogenicity realizations (Figure 4d), even though the same echogenicity realization was used with all noise realizations. This is explained as follows: The different individual SoS maps contain different speckle-related artifacts. Because the different added noise realizations lead to the exclusion of different individual SoS maps, speckle-related artifacts in the weighted average SoS map vary with added noise realization. This effect was not seen in Figure 6d because the averaging was performed over all individual maps, independent of noise realization. Figure 6h–k show corresponding results for the layer phantom. Because the same added noise was used for both digital phantoms, the standard deviation maps corresponding to Figure 6h,i are, by definition, identical to Figure 6c,d and, thus, they are not shown. The same as for the circular inclusion, the artifact level for the layer phantom is strongly reduced when excluding the corrupted maps, and the standard deviation map looks similar to the corresponding panel in Figure 4d. Thus, whereas the proposed method was shown to perform similarly to the reference method in a situation with only speckle-related artifacts, it is superior in a situation with additional noise when restricting the weighted averaging to uncorrupted SoS maps.

## 5. Discussion and Conclusions

In this study, we introduced a new SoS reconstruction method for CUTE. As opposed to simultaneously reconstructing an SoS map from all echo shift maps for different Tx/Rx angle combinations (reference method), we reconstruct an SoS map for each echo shift map individually and then calculate the final SoS map via a weighted averaging (proposed method). The weighted averaging provides a computationally efficient way to exclude corrupted data via the choice of the averaging weights, without the need to recalculate the posterior covariance matrices for each new dataset.

First, we showed that it is possible to match the regularization parameters of the two methods to achieve not only comparable spatial resolution but also similar artifact levels. The latter was confirmed both for speckle-related artifacts and for added noise-related artifacts. Therefore, there is no disadvantage in using the proposed method over the reference method. Then, we illustrated that—when excluding corrupted data—the proposed method is able to reconstruct SoS maps from noisy echo shift data with reduced artifacts compared to the reference method. This means that even if it may sometimes be difficult to identify corrupted data, it is beneficial to use the proposed method just for the sake of improved performance in cases where such an identification is possible.

To illustrate the performance of the proposed method, we chose a simplified scenario where only a part of the echo shift maps was corrupted by the added noise. This allowed us to exclude corrupted data by completely removing those maps. We chose an arbitrary fraction of five out of ten maps for this purpose. One could investigate how the fraction influences the outcome, although this would go beyond the scope of this study without adding relevant insight. In a real clinical scenario, it is often the case that all maps are corrupted; thus, eliminating full maps would not be possible.

**Figure 7:**
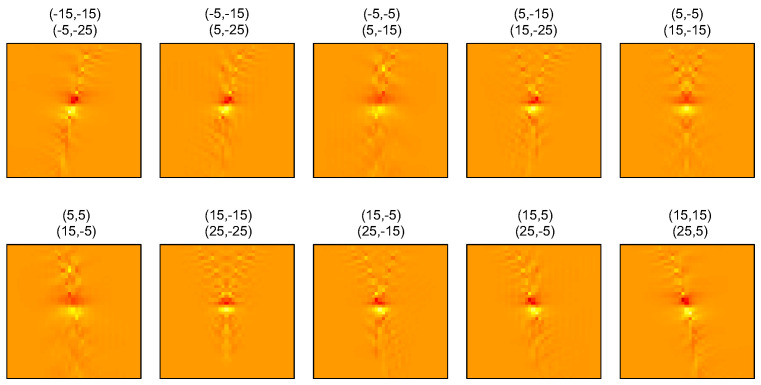
Individual SoS maps reconstructed from echo shift maps that are zero apart from one pixel in the center of the map area that was set to 0.05 μs.

One can think of various ways to determine corrupted areas within maps: (i) Identify areas directly inside the individual SoS maps. Looking at Figure 3b, it may be difficult to distinguish between artifacts and actual SoS contrast. Given that the different maps are partially redundant, real SoS contrast should look similar between the different maps. One could, therefore, identify artifacts based on the deviation of the individual maps from the average. This would, however, be difficult inside areas that are not covered by all angle combinations. (ii) Identify noisy areas inside the echo shift maps. As mentioned, in vivo echo shift noise often occurs as sparse outliers [20]. Such an appearance is not expected in a noiseless scenario and, thus, one could simply identify outliers (e.g., by using median filtering or training a deep network to do this). The challenge here is that the size of a small noisy area does not directly relate to the size of the corresponding artifact area in the SoS maps. This is illustrated in Figure 7. The individual SoS maps are shown for a hypothetical echo shift dataset, where echo shifts are zero, apart from a pixel in the middle of the map area, where the value is set to 0.05 μs. These maps show that, via Equation (7), a single noisy pixel in an echo shift map affects many pixels in the corresponding SoS map. The main influence is, however, limited to a small area around the original pixel. Areas that are identified inside echo shift maps could, thus, be translated to the SoS maps by adding a small margin. (iii) The origin of phase noise is US speckle decorrelation between Tx/Rx angle pairs. Instead of identifying noise in echo shift data, one could, thus, attempt to identify the underlying decorrelation in the CRF maps [16].

There may exist scenarios where it is not possible to find a suitable way to identify limited noisy areas. In such cases, the proposed method can be beneficial by quantifying the spatial distribution of the noise level in the different maps and designing a weighting function that optimally averages the maps, given this spatial distribution. This would likely result in an improved outcome, on average, compared to weighting all maps equally. A scenario where the proposed method has no benefit is when the noise affects the same areas in all of the maps. This is, however, not a disadvantage of the proposed method when compared to the reference method, as the latter would suffer from the same problem. In summary, we demonstrated the potential of a new method for reducing artifacts in SoS maps obtained with CUTE. Future studies will have to focus on developing concrete noise identification techniques and evaluating the performance of this technique using in vivo data.

## Data Availability

The simulation data used in this study are available upon request from the corresponding author.
